# Development of a Women's Empowerment Nurse‐Led Group Intervention for Benzodiazepine and Z‐Hypnotic Deprescription in Primary Healthcare: Results of a Co‐Creation Process

**DOI:** 10.1111/hex.70895

**Published:** 2026-10-07

**Authors:** Amaia Cuesta‐Zigorraga, Carlota Las Hayas Rodríguez, Garazi Aldanondo Aristizabal, Clara Sánchez Sánchez

**Affiliations:** ^1^ Nursing Department Faculty of Health Sciences, University of Deusto San Sebastian Spain; ^2^ Department of Medicine Faculty of Health Sciences, University of Deusto Bilbao Spain; ^3^ Deusto Physical TherapIker, Physical Therapy Department Faculty of Health Sciences, University of Deusto San Sebastian Spain; ^4^ Network for Research on Chronicity Primary Care and Health Promotion (RICAPPS) Bilbao Spain; ^5^ Biogipuzkoa Health Research Institute, Primary Healthcare San Sebastian Spain; ^6^ Osakidetza, Intxaurrondo Health Center San Sebastian Spain

**Keywords:** benzodiazepines, co‐creation, empowerment, patient engagement research, patient participation, primary healthcare, Z‐drugs

## Abstract

**Aim:**

The aim of this study was to co‐create a nurse‐led women's empowerment group intervention to facilitate the deprescription of benzodiazepines and Z‐hypnotics in primary care.

**Design:**

User‐centred design based on co‐creation.

**Methods:**

A literature review was conducted to identify female empowerment programmes that served as an initial prototype. The subsequent user‐centred design applied a co‐creation methodology in two structured phases: (1) Exploratory, to identify contextual needs and barriers, and (2) Development, to collaboratively define the intervention's final structure and content. Participants included chronic users of benzodiazepines and Z‐hypnotics and primary care professionals (doctors, nurses, and pharmacists) who actively engaged in the collaborative design process.

**Results:**

The Exploratory phase identified structural and clinical deprescribing barriers, and female clients' knowledge gaps. Empowerment was confirmed as a key facilitator. The Development phase prioritised content (self‐esteem, self‐image, and healthcare) and adjusted the group intervention's format (duration, frequency, and structure) based on participant input.

**Conclusion:**

The co‐creation process resulted in an eight‐session intervention, with support cards and a training video for nurse‐facilitators. This collaboration ensured the final design was precisely tailored to women's needs and clinical practice, maximising its feasibility and relevance. The intervention facilitates the tapering of benzodiazepines and Z‐hypnotics by combining women's empowerment with individualised and supervised dose reduction by primary care clinicians.

**What Does This Paper Contribute to the Wider Global Clinical Community?:**

Demonstrates how co‐creation with clients and healthcare professionals can lead to contextually adapted nurse‐led interventions centred on women's empowerment.

Highlights the role of women's empowerment as a catalyst for deprescribing long‐term benzodiacepines and Z‐hypnotics in primary healthcare. Introduces a structured and co‐created eight‐session intervention supported by a nurse‐training video and support cards, offering practical toolkits.

**Public Contribution:**

Clients (as users) and primary care healthcare professionals actively participated in the design of the intervention aimed at empowering women. Their involvement included participation in co‐creation workshops, in which needs and everyday realities relevant to the process of deprescribing benzodiazepines and Z‐hypnotics were identified. The structure and content of the group intervention, as well as its relevance and feasibility, were defined collaboratively. This direct participation made it possible to develop an intervention that was more client‐centred, equitable, and aligned with clinical practice.

**Reporting Method:**

This manuscript follows the short‐form Guidance for Reporting Patient and Public Involvement (GRIPP2) checklist (Staniszewska et al., 2017) to ensure transparent reporting of the co‐creation process, in which clients and healthcare professionals actively participated in the design, development, and evaluation of the intervention (Additional file 1. GRIPP 2).

## 1 Introduction

Citizen science allows non‐experts to actively participate in research under the supervision of specialised teams, facilitating more democratic, evidence‐based, and networked research [1]. Co‐creation, as a collaborative method specific to this trend, contributes to reducing the distance between science and society [2].

## 2 Background

Long‐term use of benzodiazepines and Z‐hypnotics is an important public health concern. Prolonged use is associated with tolerance, physical dependence and difficulties with discontinuation, despite recommendations to limit treatment duration [3, 4]. Discontinuation can be challenging, and evidence from primary care suggests that deprescribing interventions are more effective when dose reduction is supported by psychoeducational and behavioural strategies [5]. Such interventions may promote self‐efficacy and coping with withdrawal‐related symptoms, potentially facilitating successful discontinuation [6, 7].

Gender is particularly relevant to long‐term psychotropic medication use [8]. Women have higher levels of psychotropic medication consumption than men, and benzodiazepine use may contribute to the medicalisation of distress associated with women's social roles [9, 10]. The present study draws on a feminist and sociological perspective, which considers women's mental health and medication use in relation to social structures, gendered roles, power relations and medicalisation processes [11].

Within this framework, women's empowerment may support the recognition and mobilisation of personal resources, coping with everyday challenges and active participation in health decisions. Empowerment‐oriented approaches have been associated with improved self‐care and self‐efficacy, particularly among women [12, 13], while self‐efficacy has been identified as a relevant psychological factor in benzodiazepine deprescribing [7, 14]. Rather than assuming a direct relationship between empowerment and discontinuation, an empowerment‐oriented approach may therefore support women's engagement with individualised deprescribing.

Group‐based interventions have shown potential to foster empowerment through their inherent capacity to facilitate peer support, enable the sharing of experiences with relevant others, and provide opportunities to learn diverse coping strategies from peers [15]. Women‐only groups can offer a safe environment and facilitate approaches that consider social and contextual determinants of health [16]. In primary care, gender‐sensitive group interventions have reported improvements in self‐esteem and anxiety and depression symptoms [12, 17]. Health‐assets and salutogenic approaches similarly place individuals and their resources at the centre of care and may complement medication‐based approaches [18, 19]. This is consistent with client‐centred approaches to chronic benzodiazepine use in primary care [20].

Meaningful involvement of women in intervention development is therefore important. Client engagement promotes participation in decision‐making and integrates experiential knowledge into research and practice, consistent with the principle of ‘nothing about us without us’ and research conducted ‘by’ rather than only ‘for’ clients [21, 22, 23]. Co‐creation can challenge traditional power asymmetries in mental health care and ensure that interventions reflect participants' needs and experiences, provided that participation is meaningful and non‐tokenistic [24, 25, 26, 27].

Although psychosocial, behavioural, and multicomponent interventions have shown effectiveness in supporting benzodiazepine receptor agonist deprescribing in primary care, the available evidence is still limited for interventions specifically designed for women that are simultaneously co‐created, gender‐informed, and delivered in group formats [28, 29, 30]. This gap warrants the development of primary care interventions that combine individualised supervised tapering, non‐pharmacological psychosocial support, and shared decision‐making within a participatory framework that incorporates women's experiences, preferences, and gender‐related determinants of psychotropic drug use [31, 32]

In this context, the present study aims to co‐create a group intervention promoting women's empowerment among women who are chronic users of benzodiazepines and Z‐hypnotics, with the secondary objective of reducing their consumption. The intervention is intended to support, rather than replace, evidence‐based tapering, with dose reduction individualised and supervised by the client's primary care physician or nurse.

## 3 Methodology

### 3.1 Ethical Considerations and Data Management

The study was approved by the Research Ethics Committee of the Gipuzkoa Health Area (Ref. CUE‐EMP‐2024‐01) and complies with Law 14/2007 and the Declaration of Helsinki. Given the processing of personal data, confidentiality is guaranteed in accordance with the GDPR and Organic Law 3/2018. Data collection and analysis were conducted ethically and without recording identities, ensuring that all information disseminated was anonymous. Personal and questionnaire data were stored in separate, secure databases without identifiers, with access restricted to authorised personnel. Statistical analyses were performed exclusively with anonymised data, ensuring participants' privacy and security.

### 3.2 Design

This study is part of a larger research project whose objective is to co‐create, implement, and evaluate the effectiveness and acceptability of a group intervention led by primary care nurses, aimed at increasing female empowerment in deprescribing benzodiazepines and Z‐hypnotics.

Specifically, this study aims to describe the co‐creation process used to develop the group intervention and to report the results generated through that process. Between October and December 2024, the co‐creation procedure was conducted, the protocol for which has been previously published [33].

The process involved the participation of women who were chronic users of benzodiazepines and Z‐hypnotics, as well as the primary care healthcare professionals who regularly treat them. This study was designed as a participatory co‐creation study informed by the Participatory Action Research (PAR) paradigm. The study focused on the co‐creation of the intervention rather than on conventional qualitative data collection through individual interviews or focus groups. Qualitative data generated throughout the co‐creation workshops were analysed using Reflexive Thematic Analysis to inform the development and refinement of the intervention.

Three co‐creation workshops, each lasting approximately 2 h, were conducted. Each participant attended two workshops: an initial workshop conducted separately for healthcare professionals and female clients, followed by a joint workshop in which both groups worked collaboratively. The process was implemented sequentially across several phases (see Table 1 for a detailed description).

**Table 1:** Phases of the co‐creation process: aims, specific objectives, and implemented activities in each phase.

Phase	Aim	Specific objective	Implemented activity
Preparatory phase	Literature review & prototyping	Identify existing women's empowerment programmes and select a suitable prototype to serve as the basis for the co‐creation of an intervention aimed at supporting the effective deprescription of benzodiazepines and Z‐hypnotics in Primary Health Care.	Comprehensive review of the literature and selection of Arostegui & Martínez (2019) as the prototype.
	Participant recruitment & logistics	Establish contact with institutional gatekeepers to inform and invite the study population.	Healthcare professionals were recruited by the research team, who subsequently invited and enroled chronic BDZ/HZ users. Informed consent was obtained at the time of recruitment.
		Recruit healthcare professionals and clients, obtain signed informed consent, prepare the workshop environment and materials, develop observational data collection templates, and organise and schedule the co‐creation sessions.	The co‐creation workshop exercises and dynamics were designed and prepared in collaboration with an expert in participatory methodologies, who also led and facilitated the workshops. Templates for data collection through observation by the research team were also developed. Meeting space and materials were prepared.
Co‐creation process1) Exploratory phase	Explore pharmacological regimen	Detect, collectively and across disciplines, usual primary care interventions for BDZ/HZ deprescription and explore clients' knowledge and attitudes toward their treatment.	Technique Journey mapping: Participants are divided into small groups to explore their perceptions, knowledge, and usual practices related to the topic. Subsequently, their contributions are shared and synthesised in a collaborative conceptual map.
	Explore empowerment	Collectively define core content and objectives of a women's empowerment intervention.	Technique Journey mapping
	Explore nurse‐led group intervention	Determine collectively the characteristics of a group intervention led by nurses, including structure, temporality, and sustainability.	Technique Journey mapping
Co‐creation process2) Development phase	Develop jointly the structure and content of the intervention	Prioritise topics to be addressed in the intervention, promoting women's empowerment as a strategy for tapering BDZ/ZH.	Technique Priority matrix: small groups assign scores to topics (self‐esteem, self‐image, health self‐care, relationships, sexuality, pharmacological treatment) to determine relevance and prioritise areas.
	Develop jointly the dynamics and format of the intervention sessions	Evaluate the appropriateness of the session exercises and the overall structure of the prototype intervention.	Technique Idea laboratory: in small groups, participants rate exercises on a 1–10 smiley‐face scale for satisfaction and offer open‐ended feedback regarding the structure, format, and content of the sessions.
Co‐creation process3) Collection of data	Documentation and systematic recording of the co‐creation process	To collect, organise, and systematise qualitative and quantitative data generated during the co‐creation workshops, including participant contributions, observations, and evaluation forms.	Observational notes and field diaries completed by the research team during workshops; transcription of group discussions; compilation of feedback and ratings from exercises (e.g., priority matrix, idea laboratory) and collection of participants' evaluation forms.
Post co‐creation sessions Analysis and decision‐making	Data analysis and synthesis for prototype refinement	To analyse all data collected from workshops in order to identify common themes, validate consensus areas, and extract actionable recommendations for the design of the final nurse‐led empowerment intervention.	Thematic content analysis of qualitative data using an inductive approach; synthesis of quantitative scoring results; triangulation between researcher notes and participant feedback; discussion meetings among the research team and key stakeholders to decide on final components, structure, and implementation plan of the intervention prototype.

*Note:* For a detailed description, please refer to the protocol Cuesta‐Zigorraga and Las Hayas.^33^

Abbreviation: BDZ/ZH, benzodiazepines and Z‐hypnotics.

This approach was designed to move the co‐creation process beyond consultation towards a more equitable distribution of decision‐making power. To minimise potential power imbalances, separate Exploratory Phase workshops were held for female clients and healthcare professionals, with one workshop conducted for each group. During the joint Development Phase workshop, which brought clients and professionals together, the facilitator moderated the discussion to identify and address emerging power imbalances. These measures were intended to promote the equal consideration of female clients' perspectives alongside clinical expertise, thereby fostering a more democratic, inclusive, and collaborative co‐creation environment.

Table 1 describes the complete design phases of the co‐creation process. For a detailed description, please refer to the mentioned protocol [33].

### 3.3 Participants

The co‐creation process involved three groups of people with distinct roles: the research team, primary care healthcare professionals, and female clients who were chronic users of benzodiazepines and Z‐hypnotics. The research team facilitated, observed, documented, and analysed the co‐creation process, whereas healthcare professionals and female clients participated in the co‐creation activities. The inclusion and exclusion criteria for healthcare professionals and female clients are summarised in Table 2.

**Table 2:** The study's inclusion and exclusion criteria for clients and healthcare professionals.

Inclusion criteria	Exclusion criteria
Clients	
‐ Participants must be over 18 years of age ‐ Sex—participants whose biological sex is female are selected for this study ‐ Participants must have used benzodiazepines or Z‐hypnotics for more than 3 months	‐ Epilepsy ‐ Dementia ‐ Palliative care ‐ Open episode in the Mental Health Network ‐ Severe psychiatric comorbidity with external follow‐up in the Mental Health Network ‐ Severe psychiatric comorbidity without follow‐up in the Mental Health Network ‐ Failure to sign the informed consent form ‐ Not fluent in the language in which the intervention will be offered (Spanish)
Healthcare professionals	
‐ The staff must be actively involved in client care at the participating centre ‐ Participants must have a professional profile in family and community medicine, nursing, or primary care pharmacy	‐ Existence of conflicts of interest ‐ Failure to sign the informed consent form

Regarding the inclusion of both benzodiazepines and Z‐hypnotics users, although BZD and Z‐hypnotics target different symptoms (anxiety vs. insomnia), the sociocultural determinants underlying their chronic use in women show significant overlap [34, 35, 36], justifying a shared empowerment approach focused on the common roots of over‐medicalization.

Data saturation was not used as a criterion for determining the sample size, as the primary purpose of the study was the co‐creation and refinement of the intervention rather than the exhaustive generation of themes. The sample size was determined according to the planned composition of the co‐creation workshops, with the aim of ensuring representation of the two stakeholder groups—female clients and primary care health professionals—while maintaining group sizes that allowed for active participation and meaningful interaction. This approach is consistent with previous co‐creation studies in which group size was determined according to the objectives and interactive nature of the co‐creation process rather than statistical considerations [37, 38, 39].

Purposive sampling was used to recruit healthcare professionals and female clients. Primary care health professionals were invited to participate through institutional gatekeepers. During primary care consultations, referring healthcare professionals invited eligible female clients to participate in the co‐creation process and provided them with the participant information sheet and informed consent form. Before each co‐creation workshop, the research team contacted all participants by telephone to remind them of the session.

The health professionals and female clients were recruited at the Osakidetza Primary Care Network. In the area served by the Hernani Health Center (Gipuzkoa, Spain), the sample came from a population with relatively favourable socioeconomic indicators compared with national averages and from a health system that is publicly oriented and universally accessible. This context should be kept in mind when interpreting the findings and considering their transferability to settings with different socioeconomic or health system structures.

### 3.4 Research Team and Reflexivity

The multidisciplinary research team comprised exclusively women and included a researcher with degrees in Nursing and Psychology (ACZ), a researcher with a PhD in Psychology (CLR), and a researcher with a PhD in Pharmacology (GAA), all of whom were university teaching and research staff; a general practitioner working in the public healthcare system (CSS); and an externally contracted sociologist with a PhD and expertise in participatory methodologies. The professional backgrounds represented within the research team reflected those of the healthcare professionals involved in the co‐creation process.

The expert in participatory methodologies facilitated all sessions, while the remaining members of the research team contributed to the study design, systematic observation and data collection, data analysis, and validation of the findings.

The facilitator had no prior personal or professional relationship with the female clients or primary care health professionals participating in the co‐creation process. However, she had a contractual relationship with the research team. Participants were informed that the facilitator was a sociologist specialising in participatory methodologies, as well as of the study's aims and the rationale for the co‐creation process. She had no direct stake in the clinical issue under study, although she acknowledged an academic interest in participatory approaches and collaborative intervention design.

The multidisciplinary composition of the research team and the researchers' different professional perspectives were considered as part of the reflexive analytical process. The researchers' professional backgrounds informed their perspectives on the topic under study and may have influenced both the co‐creation and data interpretation. The nursing and medical perspectives brought a focus on primary care practice and the clinical management of benzodiazepine and Z‐hypnotic use; the psychology perspective contributed attention to women's psychological well‐being and strategies of coping; the pharmacological perspective informed consideration of medication use and deprescribing; and the sociological perspective contributed a focus on gender, social context, power relations, and participatory processes.

These disciplinary perspectives may have shaped which topics and issues were considered relevant during the co‐creation process, the questions and areas explored with participants, and the researchers' interpretation of participants' contributions. The researchers therefore recognised that their professional roles and assumptions could influence both data collection and analysis, including which aspects of participants' experiences were foregrounded or interpreted as particularly salient. Rather than treating researcher subjectivity as a source of bias to be eliminated, it was understood as an integral part of the interpretive process. Reflexivity was promoted through critical discussion of emerging interpretations, consideration of alternative perspectives, and ongoing reflection on how the researchers' professional backgrounds, roles, and interactions with participants could shape the co‐creation process and subsequent data interpretation.

To promote equitable participation and minimise undue researcher influence, the facilitator used moderated discussion, encouraged participants who had contributed less to express their views, redirected the discussion when some participants dominated, and ensured that differing perspectives were explored without prioritising professional perspectives over clients' experiences. The separate Exploratory Phase workshops also provided each stakeholder group with an opportunity to express their views independently before the joint discussion. When disagreements arose, the facilitator encouraged participants to explain the reasoning behind their views and supported the group in identifying areas of convergence while acknowledging differences where consensus was not reached.

### 3.5 Data Collection Techniques

Data were collected using several techniques aligned with the different phases and objectives of the co‐creation process (see Table 1). These techniques were selected to capture different types of information, including participants' experiences, perceptions, knowledge, needs, opinions, priorities, suggestions, and evaluations regarding benzodiazepine and Z‐hypnotic use, deprescribing, empowerment, and the proposed intervention.

No audio or video recordings were made. Given the participatory and collaborative nature of the workshops, recording was considered potentially disruptive to natural interaction and spontaneous participation. Instead, trained members of the research team systematically documented the sessions using structured observation templates and detailed field notes.

‐ **Structured observation templates.** Structured observation templates were completed by members of the research team during each workshop exercise to systematically document participants' contributions, interactions, and relevant observations (Additional File 2. Information collection template). The templates were used to record information related to the topics addressed in each activity, including participants' experiences, perceptions, knowledge, opinions, needs, and suggestions. They also captured relevant aspects of group interaction and participation.
‐ **Field notes.** Field notes were used to record additional contextual observations and reflections arising during the workshops. These notes complemented the structured observation templates by documenting relevant aspects of the discussions, group dynamics, interactions between participants, and contextual information that could contribute to the subsequent interpretation of the data.
‐ **Journey mapping.** Journey mapping was used as a participatory visual technique in each workshop with both healthcare professionals and female clients. Participants worked in small groups to discuss and explore their perceptions, experiences, knowledge, needs, and practices related to three key areas: the benzodiazepine and Z‐hypnotic deprescribing process, the conceptualisation of empowerment, and the suitability of the proposed intervention conditions. Participants recorded their contributions on Post‐it notes, which were then collected, reflected back, and organised visually by the facilitator to construct collaborative journey maps. The maps captured different stages and aspects of the deprescribing journey, including perceived barriers, needs, facilitators, and intervention requirements. The resulting maps and accompanying discussions were subsequently analysed and synthesised as part of the qualitative analysis.
‐ **Priority matrix.** The priority matrix was used during the joint workshop with all participants, including female clients who were chronic users of the drugs under study and healthcare professionals. Participants were divided into small mixed groups of four to five participants. The aim of the activity was to collectively identify and prioritise the objectives and content considered most relevant for an intervention aimed at promoting female empowerment and supporting the deprescribing of benzodiazepines and Z‐hypnotics. The activity was facilitated by the expert in participatory methodologies. A total of 100 small stickers, representing 100 points, were distributed among the groups, with each group receiving a proportion of the total. Participants were asked to allocate the stickers among the predefined topics according to the importance collectively attributed to each area, placing them on a large panel displaying the different topics. The topics presented to all groups were self‐esteem, self‐image, pharmacological treatment, gender‐based violence, couple relationships, sexuality, motherhood, and healthcare. Participants discussed the relevance of each topic within their group and reached a collective agreement on how to distribute the stickers. The scores assigned to each topic across the groups were subsequently compiled to determine their relative priority. The activity therefore generated quantitative information on the relative importance assigned to each topic, complemented by qualitative information from the group discussions regarding the reasons underlying participants' priorities. This information was used to inform the subsequent co‐creation process and guide the selection and emphasis of the content to be incorporated into the intervention.
‐ **Idea laboratory.** The idea laboratory was conducted during the joint workshop to assess and refine the content, structure, format, and perceived usefulness of the proposed intervention sessions. Four mixed subgroups, each comprising female clients and healthcare professionals, evaluated the sessions included in the prototype intervention developed by Arostegui Santamaría, Martínez Redondo [16]. The original programme comprised 13 sessions; however, the final session, which was considered a closing session, was excluded from the co‐creation evaluation. Consequently, the 12 remaining sessions were distributed across the four subgroups, with each subgroup evaluating three sessions, ensuring that all sessions were assessed.

For each assigned session, participants first reviewed its proposed objectives, content, and activities and discussed their suitability within the subgroup. They then assessed the usefulness of the proposed dynamics or exercises using the question: “Is the proposed dynamic or exercise useful for achieving the objectives set for the session?” Participants rated the proposed activities on an ordinal scale from 0 to 10, where 0 indicated the lowest and 10 the highest perceived usefulness. They subsequently evaluated the structure and format of the session in terms of its suitability, organisation, and ability to facilitate active and effective participation aimed at empowering women. Participants were encouraged to justify their ratings and identify potential modifications to the content, activities, timing, organisation, or format.

One member of the research team was assigned to each subgroup and facilitated the evaluation process. Before each evaluation, the researcher briefly introduced the objectives and content of the session under review and explained the evaluation procedure. The researcher facilitated the discussion without directing participants towards particular responses and systematically recorded quantitative ratings, qualitative comments, suggestions, and proposed modifications using a structured data collection template. Quantitative ratings provided information on participants' assessment of the proposed activities, while qualitative discussions and comments informed their refinement.

**Self‐reported questionnaire.** A self‐reported questionnaire was developed ad hoc by the research team and administered to all participants. The questionnaire comprised nine items: seven assessed participants' satisfaction with the participatory methodology, including their perceptions of the information received about the project, the methodology used, their participation in the co‐creation process, and the perceived usefulness and effectiveness of the process; two additional items assessed the perceived validity of the content of the proposed empowerment intervention. All items were assessed using a five‐point Likert‐type scale ranging from ‘strongly disagree’ to ‘strongly agree’. Sociodemographic characteristics were collected from female participants only and were used to characterise the client sample. Questionnaire data were incorporated into the quantitative analysis.

No transcripts or session notes were returned to participants for comment, as data collection focused on collaborative co‐creation activities rather than individual interviews.

### 3.6 Data Analysis

For the qualitative data generated during the co‐creation sessions, we applied Reflexive Thematic Analysis (RTA), following Braun and Clarke [40, 41]. The analysis followed an inductive and iterative approach, through which codes and themes were developed through active engagement with the data and consideration of the contextualised perspectives of participants.

The data were initially coded by ACZ through a systematic engagement with the workshop notes and collaborative maps. The developing codes and interpretations were subsequently discussed with the research team, allowing alternative perspectives to be considered and the themes to be progressively refined. These discussions aimed to deepen the interpretation of the data rather than to reach consensus on a single correct coding.

For the quantitative data collected using a self‐reported questionnaire, we calculated descriptive statistics such as means, standard deviations, and response frequencies. We used R statistical software for the statistical analyses.

## 4 Results

The results are structured according to the different phases of the co‐creation process outlined in Table 1.

### 4.1 Preparatory Phase

#### 4.1.1 Literature Review and Prototyping

In the first step of the process, a targeted literature review was conducted to identify existing women's empowerment programmes that could serve as potential prototypes for the co‐creation of an intervention aimed at supporting the effective deprescribing of benzodiazepines and Z‐hypnotics in primary care. The literature review and selection of the prototype were conducted by the research team. Existing women's empowerment programmes were identified and reviewed according to their relevance to women's empowerment, gender perspective, target population, and applicability to the primary care context. The identified programmes were discussed collectively by the research team, and the programme considered most appropriate for adaptation through the co‐creation process was selected. As the purpose of the review was to identify a suitable intervention prototype rather than to assess the effectiveness of the identified programmes, no formal methodological quality assessment was undertaken. The team reviewed existing women's empowerment programmes and jointly selected the Manual for Relapse Prevention with a Gender Perspective by Arostegui Santamaría, Martínez Redondo [16] as the most appropriate prototype for the co‐creation process. This manual places gender at the centre of addiction intervention and proposes creating a space for female empowerment that prioritises a social—rather than exclusively biological—and feminist understanding of health. The authors articulated their proposal in 13 working sessions. These sessions were aimed at strengthening women's self‐esteem and empowerment in relation to their treatment. They also sought to promote the recognition of gender mandates as determinants of health. In addition, they aimed to promote emancipation using health assets and to address gender‐based violence from a sociocultural perspective, highlighting its impact on mental health.

This programme was chosen because it explicitly integrates a gender approach and addresses key psychosocial factors in the prolonged use of psychotropic drugs. The detailed description of the 13 sessions and proposed activities provided a clearly structured prototype that could be adapted and refined through the co‐creation process. This was particularly relevant because it allowed us to address the use of psychotropic drugs in relation to the structural inequalities that affect women's lives, as well as coping strategies conditioned by gender mandates, thus facilitating a more comprehensive intervention that is sensitive to the sociocultural context.

#### 4.1.2 Participants

The group of healthcare professionals (*n* = 10) comprised nine women and one man: two primary care pharmacists, four primary care nurses, and four family and community doctors.

Seven women with chronic use of benzodiazepines and Z‐hypnotics participated, and their sociodemographic characteristics are summarised in Table 3. All were Spanish nationals; however, during the recruitment process, an effort was made to reflect the diversity of origins present in the community where the co‐creation took place. In this regard, one participant was a migrant, and the others were native. Economic situation was assessed based on participants' self‐perception, reflecting their subjective assessment rather than an objective measure of income.

**Table 3:** Sociodemographic characteristics of participating female clients (*N* = 7).

Variable	*n*	%	Variable	*n*	%
Age group (years)			Level of education		
30–49	2	28.6	None or Primary Education	2	28.6
50–69	3	42.9	Secondary Education	1	14.3
70 or older	2	28.6	Vocational Training or University	4	57.1
Marital status			**Economic situation**		
Single	2	28.6	Very good	0	0.0
Married or in a relationship	3	42.9	Good	3	42.9
Widowed	1	14.3	Fair	3	42.9
Separated or divorced	1	14.3	Poor	1	14.3
Employment			Very poor	0	0.0
Active, still working	4	57.1			
Retired or pensioner	3	42.9			

Regarding participation, no participants initially refused to take part; partial attendance was due to scheduling conflicts. Of the 17 participants, five attended just one of the two designated workshops—four healthcare professionals and one female client.

No one else besides the participants and the research team was present during the co‐creation workshops. No repeat interviews were conducted; each participant took part in only one session per workshop.

### 4.2 Results of the Co‐Creation Workshop Activities

As described in the Design section, several participatory activities were conducted during the co‐creation workshops. The following paragraphs present the results generated during the Exploratory Phase. The outputs were analysed to identify themes and categories emerging directly from participants' contributions. For illustrative purposes, the main themes are presented alongside exemplary workshop data, while minor themes and case‐specific variations are not reported.

#### 4.2.1 Exploratory Phase

##### 4.2.1.1 Tool: Journey Mapping

The findings from the healthcare professionals' workshop are presented first, followed by those from the female clients' workshop, as the journey mapping activities were conducted using the same approach in both groups. The information collected through the journey maps developed by each group was synthesised and is presented in Figure 1 and Figure 2, respectively.

a. **Healthcare Professionals.** The group interactions were collaborative. Participation was unbalanced, as some professionals took on a predominant role in the interventions, making it necessary to encourage debate to balance the contributions. The level of consensus varied depending on the topic discussed, reflecting differences in the participants' perspectives and experiences.

**Figure 1:**
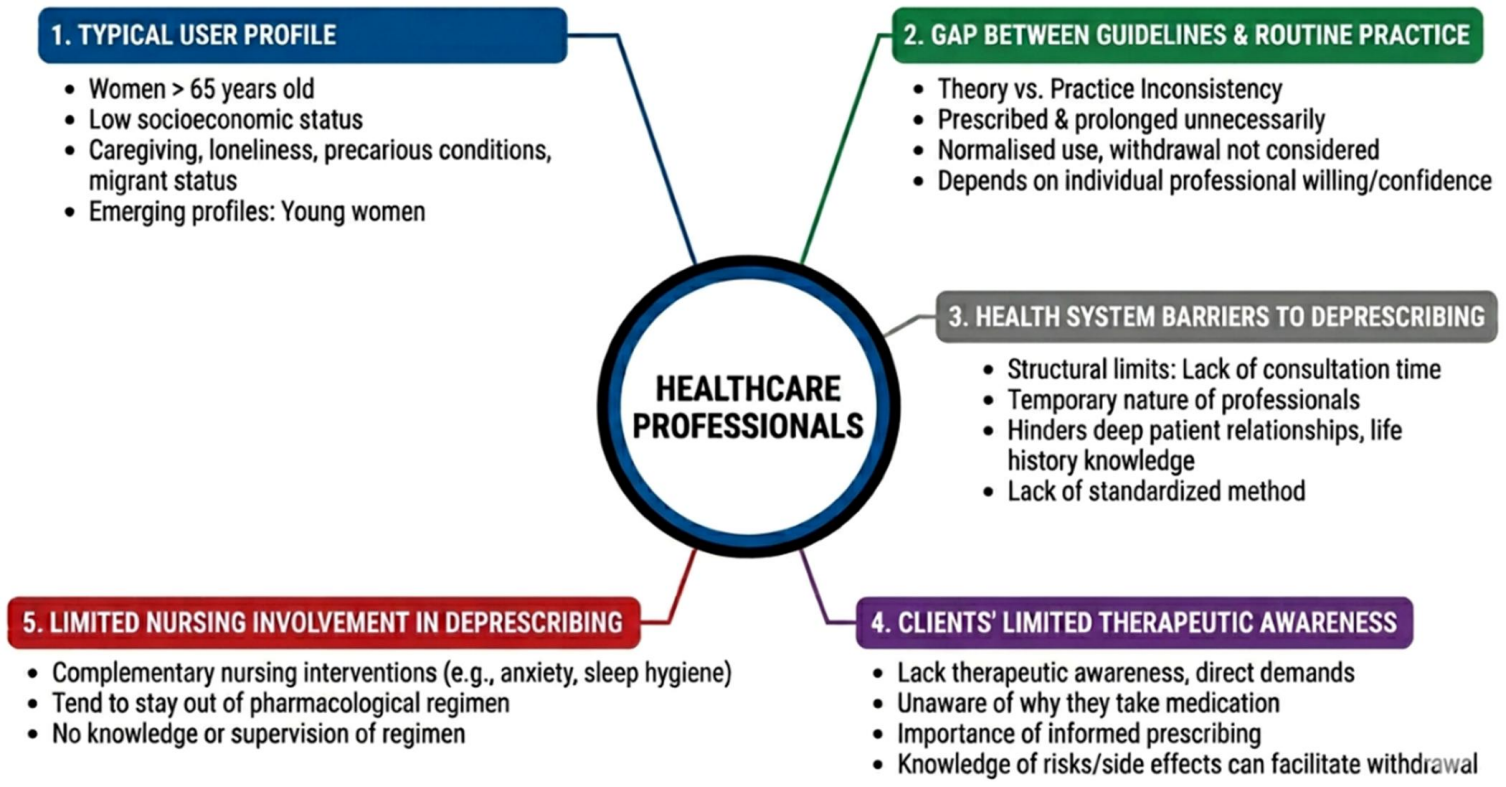
Conceptual framework of journey mapping results: Healthcare professionals' perspectives in deprescribing in Primary Care.

**Figure 2:**
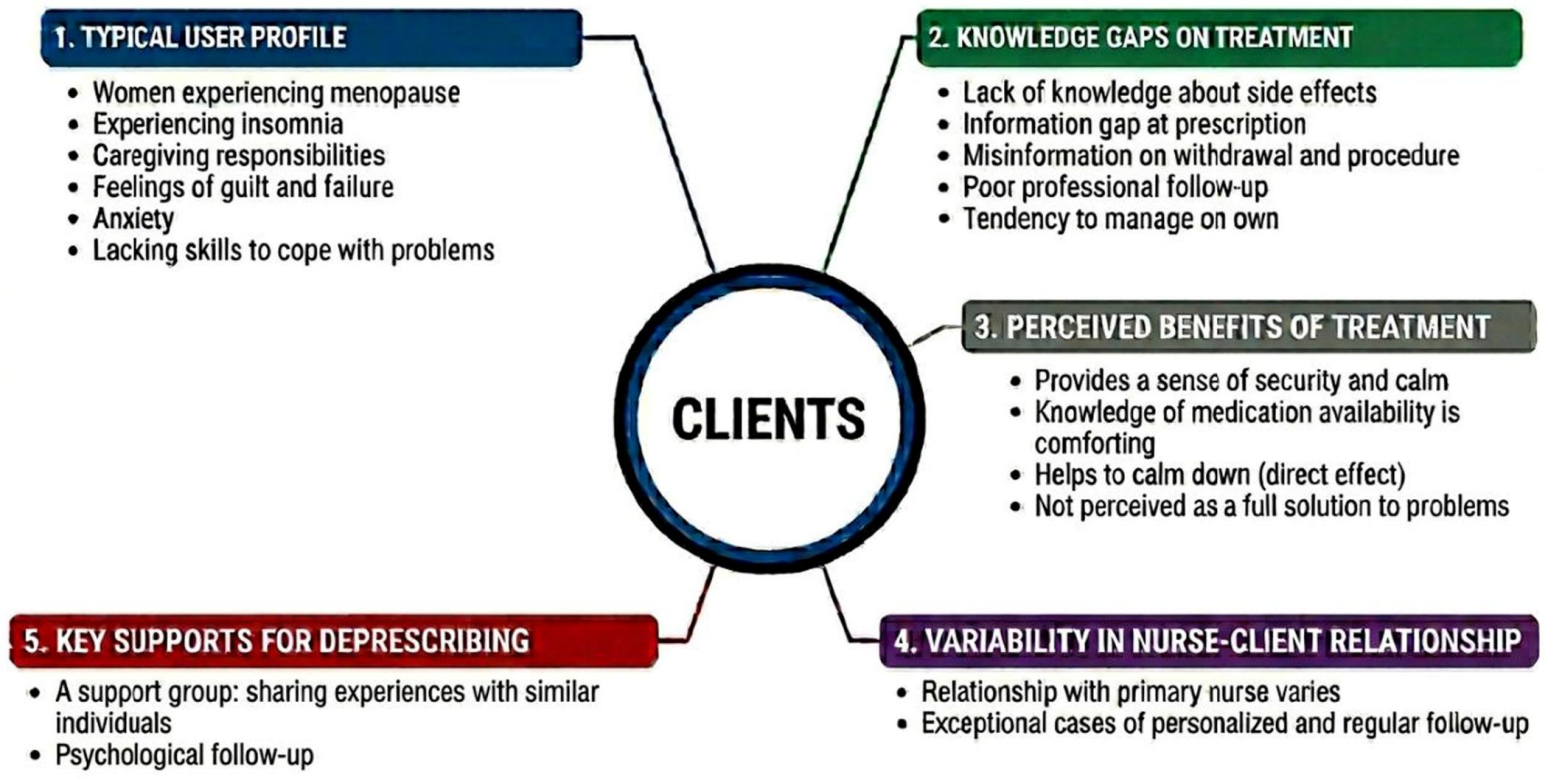
Conceptual framework of journey mapping results: Female clients' perspectives on deprescribing in primary care.

###### 4.2.1.1.1 On the Deprescribing Procedure

i. Typical User Profile
  Healthcare professionals identified the profile of clients who chronically use benzodiazepines and Z‐hypnotics as women over 65 years old with a low socioeconomic status. One participant corroborated some of the characteristics mentioned by the other members: ‘*I have consulted the profile of the outpatient clinic's patients, and most are women over 65 years of age and on monotherapy*’ (Pharmacist). Overburdening by caregiving, loneliness, difficulties in achieving work‐life balance, precarious living conditions, and migrant status were highlighted as everyday problems that motivate the use of these drugs. The emergence of new profiles among young women was also noted.
ii. Gap Between Guidelines and Routine Practice
  It is recognised that routine practice differs from clinical recommendations, and that ‘these medications are prescribed when they should not be and are prolonged when they should not be’. Similarly, ‘the use of these drugs is normalised, and withdrawal is not considered because it is not given importance. We all know it should be done, but it is not’. It is perceived that monitoring medication regimens and considering withdrawal ‘depends on the confidence and willingness of each professional’.
iii. Health System Barriers to Deprescribing
  The lack of time during consultations and the temporary nature of the healthcare professionals involved were identified as the main structural limitations that favour the prescription of benzodiazepines and Z‐hypnotics and hinder their withdrawal. There was broad consensus on the importance of knowing the client's life history and ensuring continuity of care, as ‘interventions depend on the relationship with the patient’. Understanding the client's life history and the cause of their emotional problems facilitates the establishment of a pharmacological regimen adapted to their needs, as well as the identification of the appropriate time to consider deprescribing. In relation to this, the following conclusion was reached: ‘the less I know about the patient's life history, the more I prescribe’. It was also noted that knowing the cause of the problem requires commitment, and ‘it is easier to prescribe and not investigate’.
  Finally, regarding the tools available for deprescribing potentially inappropriate regimens, healthcare professionals are aware of them but recognise that ‘*a standardised method is lacking*’ in routine practice.
iv. Clients' Limited Therapeutic Awareness
  As for clients, family doctors often deal with users who lack therapeutic awareness and sometimes make direct demands (‘give me Orfidal; give me something to sleep’). ‘Many patients are unaware of why they are taking the medication; they take it because they have been doing so all their lives’. In this regard, the importance of informed prescribing was highlighted since the client's knowledge of the risks and side effects associated with consumption (as well as their experience of them) acts as a facilitating factor for drug withdrawal: ‘I have seen patients request withdrawal when there is news that the drugs produce side effects’. One nurse added the following nuance: ‘When prescribing, it would be important to inform patients that it is not forever, that they have to work on the underlying issues, that the medication is temporary’.
v. Limited Nursing Involvement in Deprescribing
  Community nursing increasingly incorporates anxiety management and sleep hygiene recommendations as complementary strategies to pharmacological treatment. In general terms, it was observed that nurses tend to stay out of the pharmacological regimen, without getting involved in its knowledge or supervision. In relation to this, assistant nurses stated: ‘If I see a woman who has been taking this medication for a long time, I overlook it; I don't consider saying anything to the doctor. What I suggest is adding some extra help’; ‘I have never seen or participated in a deprescription, nor do I consider reviewing the patient's pharmacological regimen’.

###### 4.2.1.1.2 On Understanding the Term ‘Empowerment’ and Its Relevance

Healthcare professionals defined empowerment as having the necessary skills to cope with everyday life and relying on one's own resources to solve daily challenges independently, as ‘the power of the self’. It was considered a desirable and facilitating condition for successful drug withdrawal.

###### 4.2.1.1.3 On the Adequacy of the Conditions for Intervention

i. Inclusion Criteria for Participation in the Empowerment intervention
  There was a high level of consensus among participants in defining the criteria for access to the therapeutic group to promote female empowerment in primary care. In this regard, clinical stability in emotional symptoms, motivation and confidence in the process, awareness of the problem, and willingness to share experiences were considered necessary conditions for access. On the contrary, situations such as cognitive impairment, being at a critical juncture in life, suffering from a severe mental disorder, or being monitored by the Mental Health Network were evaluated as contraindications.
ii. Nurses as Group Leaders
  Regarding leadership of the group intervention, nurses expressed uncertainty about taking on this role with their current knowledge and indicated, with a high level of consensus, that ‘*to apply such an intervention, they need specific training on how to facilitate a group*’. On the other hand, a family doctor supported by her colleagues in the same profession indicated that, despite the doubts, nursing was the appropriate group to lead the intervention: ‘*nurses have a biopsychosocial approach to health and are the ones who know the different areas of the patient's life*’. This intervention does not constitute psychotherapy, but rather a structured self‐management programme focused on patient safety and the promotion of personal resources.
iii. Intervention Format
  Issues related to the intervention's timing and structure generated diverse opinions. In terms of frequency, it was considered appropriate to propose a group intervention for female empowerment from the health centre with a frequency that could vary between weekly and monthly, with each session lasting 1–2 h. It was determined that the optimal number of sessions for the intervention ranges from six to eight. Regarding the format, although face‐to‐face sessions were prioritised, some participants suggested incorporating a virtual platform as a complement. This option would enrich the experience and facilitate access to the content. In addition, it would allow those who prefer not to participate in a face‐to‐face group to benefit from the intervention.

b. **Female Clients.** Although the participants arrived with doubts about the workshop's purpose, expressing uncertainty about their role, the group showed dynamism in the proposed exercise, with a high level of participation. Interactions were collaborative, and participation was balanced and flowed naturally. On occasions, it was necessary to redirect the discussion because it drifted towards personal experiences that were irrelevant to the study's objective. The level of consensus among participants was high.

###### 4.2.1.1.4 On the Deprescribing Procedure

i. Typical User Profile
  The clients described the profile of a regular user of benzodiazepines and Z‐hypnotics as a woman experiencing menopause, insomnia, caregiving responsibilities (especially related to her children), feelings of guilt and failure, and anxiety, as well as lacking skills to cope with problems and concerns.
ii. Knowledge Gaps on Treatment Adverse Effects and the Deprescription Procedure
  Regarding perceptions of the pharmacological regimen, a lack of knowledge about its side effects was highlighted due to the absence of information at the time of prescription: ‘They didn't explain anything about the side effects to me, and I've been taking it for years’. Similarly, misinformation was detected regarding the clinical indications for withdrawal and the procedure for doing so. Along the same lines, the participants showed broad consensus that follow‐up of pharmacological treatment by healthcare professionals is poor: ‘Nobody has called me, there is no follow‐up’. Some of the women said that, as a result of the situation, they tend to ‘manage on their own’.
iii. Perceived Benefits of Pharmacological Treatment
  Attitudes towards the pharmacological regimen were expressed as follows: ‘It calms me down, but not much else’ and ‘It's not the solution to my problems, but it helps me’. Several participants highlighted the sense of security and calm that knowing they have the medication available gives them (‘It helps me to know that I have it in my bag’), beyond its direct effect.
iv. Variability in the Nurse–Client Relationship
  Perceptions varied regarding the relationship with their primary nurse at the health centre. There were exceptional cases of personalised and regular follow‐up (‘My nurse knows me very well and advises me’), which positively surprised the rest of the participants. However, most clients reported that the nurse at their health centre was unfamiliar with the more profound aspects of their lives and even stated: ‘I don't know who my nurse is’.
v. Key Supports for Deprescribing
  During the discussion, two fundamental pillars were identified as aids to deprescribing benzodiazepines and Z‐hypnotics: the support group and follow‐up by a psychologist. The support group was considered appropriate because it allowed participants to ‘share my experiences with other people in a similar situation to mine’ and because ‘it helps us to share and listen to things that work for each other’. The power of ‘sharing’ in their daily lives was also highlighted as a way of not feeling alone with their problems (‘It has helped me a lot to talk to people to see that you are not the only person and that many people are in the same situation as you’). On the other hand, there was broad consensus that ‘if you take these pills, you have to have psychological follow‐up’. In this regard, the support offered in their care setting was assessed as deficient, since ‘they don't teach you how to manage it’.

###### 4.2.1.1.5 On Understanding of the Term ‘Empowerment’ and Its Relevance

Clients described empowerment with expressions such as ‘believing in yourself’, ‘having the freedom to choose’, and ‘having self‐esteem’. These issues were considered relevant when facing the challenges of everyday life; therefore, empowerment was recognised as a favourable condition for dispensing with drugs such as benzodiazepine and Z‐hypnotics.

###### 4.2.1.1.6 On the Adequacy of the Conditions of the Intervention

i. Design of the Nursing‐Led Group Intervention
  The following describes the characteristics that the participants collectively defined for a group intervention led by primary care nurses, aimed at facilitating adherence. It was agreed that heterogeneity in sociodemographic profiles (in terms of age, origin, and social class, e.g.) fosters an enriching group environment and enhances collective learning. Therefore, it was jointly agreed that it would be most appropriate to target the group intervention ‘at all women, without restrictions’ and to propose it in an open format, where participation is flexible. In other words, ‘the door should always be open’, and ‘each person should be allowed to decide when to attend, having been informed that the resource exists’ at their local health centre. However, the importance of sharing the common denominator of ‘taking the same pills’ was emphasised. As for the format, there was broad consensus that the appropriate duration would be 1.5 h per session, with face‐to‐face meetings monthly (‘every 15 days would be too much for me’; ‘I know that once a month, I will be able to attend’). The suitability of nurses to lead a women's empowerment group generated diverse opinions, and a psychologist role was proposed for this purpose: ‘maybe a nurse is fine, but maybe it could be a psychologist’. In any case, interpersonal skills such as closeness, empathy, and active listening (‘someone who knows how to listen’) were considered essential for leading a support group. Similarly, a humanised, non‐judgmental approach and specialised training were considered necessary to facilitate a group of this nature.

#### 4.2.2 Development Phase

This section presents the results of the participatory activities conducted during the joint workshop of the Development Phase. These activities focused on evaluating and refining the content and structure of the prototype intervention, as well as prioritising the topics to be addressed.

a. Tool: Priority Matrix
  The prioritisation results obtained through the priority matrix are presented in Figure 3. Across the mixed groups, the 100 points were allocated among the eight predefined areas according to their perceived importance for promoting female empowerment and supporting the deprescribing process. Overall, self‐esteem, self‐image, and healthcare received the highest number of points and were therefore identified as the highest‐priority areas. These were followed, in descending order of priority, by motherhood, gender‐based violence, couple relationships, and sexuality, whereas pharmacological treatment received the lowest number of points.
b. Tool: Idea Laboratory
  The Idea Laboratory generated quantitative and qualitative information on participants' assessment of the prototype intervention sessions. Quantitative ratings reflected the perceived usefulness of the proposed activities, while qualitative feedback provided information on the suitability, structure, format, and acceptability of the sessions, as well as suggestions for improvement. The research team reviewed and synthesised this information to identify aspects of the prototype sessions that required modification, elimination, or further development during the co‐creation process. The resulting changes and the corresponding feedback from participants are presented in Figure 4 and detailed in Additional File 3 (Results: Idea Laboratory).

**Figure 3:**
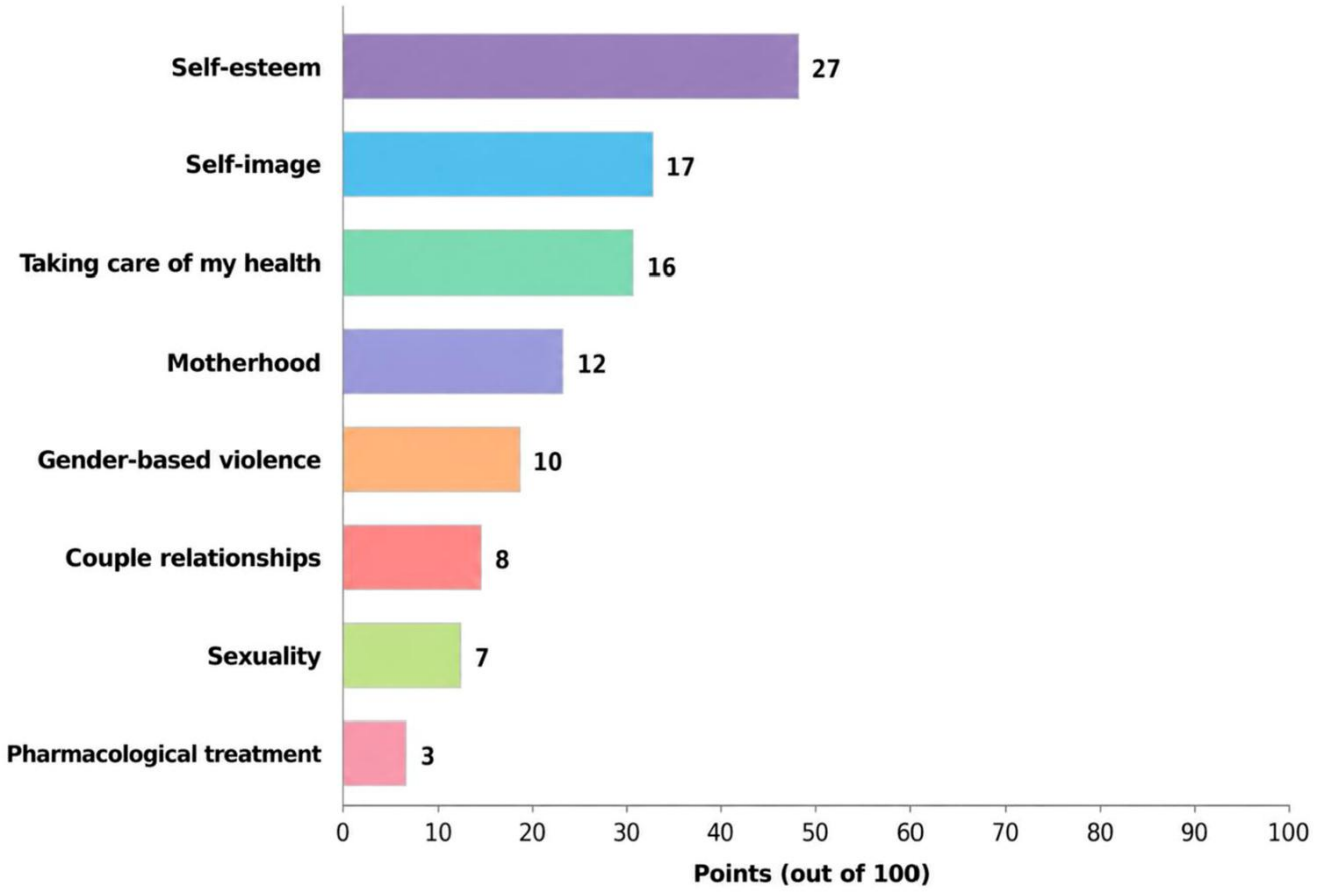
Priority ranking of topics for a women's empowerment intervention based on healthcare professionals' and female clients' evaluations using the priority matrix.

**Figure 4:**
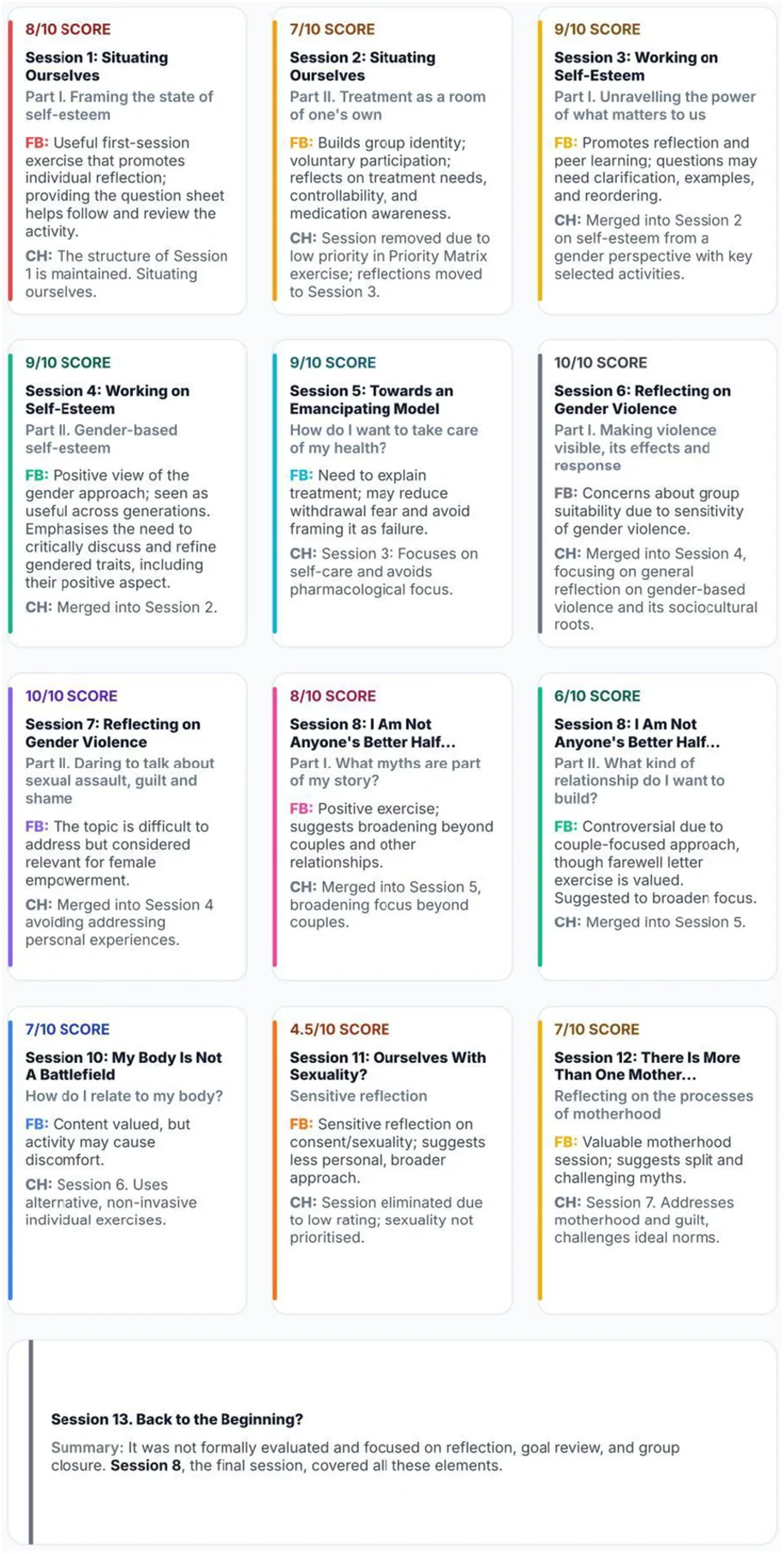
Evaluation of the prototype intervention content through the ‘idea laboratory’ exercise with mixed groups of female clients and healthcare professionals. Session scores are represented on a 10‐point scale. CH indicates implemented changes; FB, participant feedback.

#### 4.2.3 Clinical Safety and Referral Pathway

Clinical safety emerged during the co‐creation process as a cross‐cutting requirement for the intervention. Participants highlighted the need to distinguish the empowerment and peer‐support components from the clinical management of medication tapering. The role of the nurse facilitator was identified as an important safeguard for monitoring participants' wellbeing and recognising situations requiring clinical assessment. The co‐created protocol therefore identified potential ‘red flags’, including paradoxical reactions, severe rebound insomnia, and signs of psychological decompensation, as situations requiring escalation to clinical care. Based on these contributions, a referral pathway was incorporated into the intervention design, whereby participants presenting a red flag would be referred to their primary care physician or reference nurse for individualised clinical assessment.

### 4.3 Participants' Perception and Satisfaction

Of the 17 people who participated in the workshops, 16 completed the self‐reported satisfaction questionnaire. The results are presented in Figure 5.

**Figure 5:**
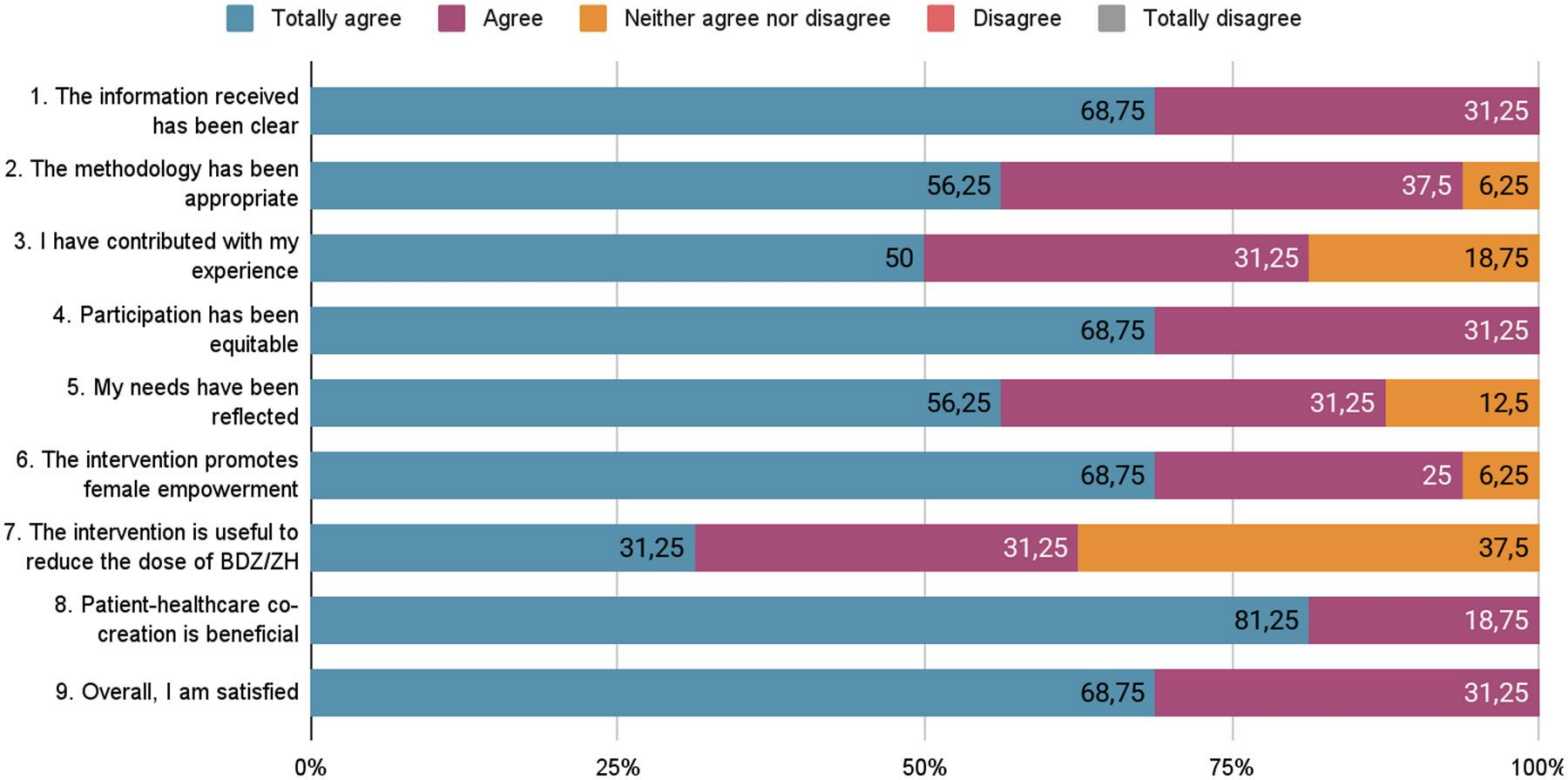
Results of the self‐reported questionnaire for the 17 participants, including healthcare professionals and clients.

Overall, participants reported a high level of satisfaction with the co‐creation process. Most responses were positive, suggesting that participants perceived the methodology as appropriate and valued the opportunity to contribute to the development of the intervention. However, some neutral responses were observed, particularly for Q7. These responses indicate that, although the co‐creation process was positively perceived, the findings should not be interpreted as evidence of the feasibility or effectiveness of the resulting intervention.

### 4.4 Final Intervention and Components

Based on the findings generated throughout the co‐creation process, the final intervention consisted of eight group sessions led by primary care nurses, complemented by a training video and session‐specific support materials. The intervention promotes women's empowerment by addressing areas such as self‐esteem, self‐efficacy, self‐image, and self‐care within a shared space where women can exchange experiences and support one another. It is designed to accompany and facilitate the individualised reduction of benzodiazepines and Z‐hypnotics.

- **Training video:** An 18‐min training video was developed as a complementary resource to support primary care nurses in facilitating the group intervention. The video is structured into two main components. First, it provides practical guidance on group facilitation, with particular attention to creating a safe, respectful, and non‐judgmental environment for women undergoing a potentially vulnerable process such as medication deprescribing. It addresses strategies to promote equitable participation, active listening, empathy, confidentiality, and trust, as well as approaches to managing disagreement and behaviours that may hinder group cohesion and psychological safety. Second, the video provides practical demonstrations of the exercises proposed for each intervention session. The activities are simulated to offer nurses a visual example of how to introduce, explain, and facilitate each exercise, thereby supporting intervention delivery while allowing adaptation to the characteristics and needs of each group. Additionally, the video explains how to use the support cards during the intervention sessions.
- **Support cards:** A set of session‐specific support cards was developed for nurses, with one card corresponding to each intervention session. Each card provides the essential information required to facilitate the group, including the session objectives, a structured session plan describing the proposed exercises, the materials required, and practical guidance and key considerations for effective group facilitation. These cards are intended to provide nurses with a concise and practical reference during each session while allowing sufficient flexibility to adapt the activities to the needs and dynamics of each group.

For further details, two screenshots from the training video are provided in the Supplementary Material, illustrating the guidance for appropriate group facilitation and the use of the support cards.

## 5 Discussion

This study successfully co‐created a nursing‐led intervention using a participatory, client‐centred approach. The findings informed the development of a nursing‐led intervention tailored to the needs and perspectives of key stakeholders.

### 5.1 Chronic Use Profiles and Determinants

The workshop findings were consistent with evidence showing that chronic use of benzodiazepines and Z‐hypnotics is prevalent among older women and increases with age (Ministry of Health, 2024; Subdirección General de Información Sanitaria [42]), with limited therapeutic alternatives contributing to prolonged use [43]. Participants also identified increasing consumption among younger women, potentially reflecting gender inequalities from earlier stages of life [44].

Emotional distress, internal conflicts, and social pressure were identified as relevant factors, consistent with previous research [45, 46]. Previous studies have associated being divorced, widowed, unmarried, or living alone with an increased likelihood of psychotropic medication use, including benzodiazepines and, particularly, long‐term benzodiazepine use. However, these associations may be partly explained by underlying mental health conditions, socioeconomic disadvantage, and reduced social support [47, 48, 49]. These findings support considering the social and emotional context of medication use in deprescribing interventions, as reflected in the intervention developed through co‐creation.

### 5.2 Barriers and Enablers for Safe Deprescribing

Primary healthcare professionals identified barriers consistent with previous research, including healthcare pressure, insufficient longitudinal follow‐up [50, 51], limited professional training on prolonged use (Hürlimann et al., 2024), and insufficient accessible information about withdrawal [52, 53]. These findings informed the development of a nursing‐led group intervention incorporating a training video and professional support materials to strengthen preparation and information throughout the deprescribing process.

### 5.3 The Role of Empowerment in Medication Reduction

Empowerment was considered an important intervention component, although healthcare professionals emphasised autonomy‐building tools while clients prioritised self‐esteem and decision‐making. Consistent with Rappaport [54], empowerment may support women's control over decisions affecting their lives. The co‐creation process therefore positioned empowerment alongside, rather than secondary to, medication reduction. The high satisfaction reported by participants further supports its relevance within the resulting intervention.

### 5.4 Psychotherapeutic Interventions in Primary Care

Previous evidence suggests that brief educational interventions may have limited long‐term effectiveness, while cognitive‐behavioural approaches have shown more favourable abstinence outcomes in some studies [5]. Multicomponent approaches appear to be more effective than single‐component interventions, although the certainty of evidence remains low [30]. This supports incorporating psychosocial and educational components into deprescribing interventions. Participants also prioritised a women‐only group format, which may promote cohesion and facilitate discussion of gender‐sensitive issues such as motherhood, self‐esteem, and violence [16, 55, 56]. Accordingly, the resulting intervention combines medication reduction with emotional regulation strategies addressing discomfort associated with gender mandates.

### 5.5 Characteristics of the Co‐Created Empowerment Intervention

Stakeholder perspectives informed participant selection, leadership, and format. Healhcare professionals prioritised emotional stability, whereas clients valued group heterogeneity [57]. Although clinical exclusions remain necessary in cases of cognitive impairment [58], strict motivation criteria were softened to support programme viability and accessibility.

Nursing leadership was supported by participants' perspectives and evidence highlighting community nurses' comprehensive approach and continuity of care [59, 60], as well as their previous involvement in interventions reporting high abstinence rates [61]. Participants selected face‐to‐face 90‐min sessions to promote participation, adherence, and group cohesion, consistent with group psychotherapy recommendations [62, 63, 64].

### 5.6 Core Content and Group Dynamics

Participants prioritised gender‐perspective reflection and social support networks as key elements of psychosocial empowerment [65]. They also highlighted the importance of addressing the emotional meanings of medication use [66] and its sociocultural context [10]. Sensitive issues, including motherhood‐related guilt [67] and gender‐based violence [68], were introduced gradually throughout the intervention to ensure that these topics could be addressed in a way that respected participants' privacy and emotional safety.

### 5.7 Co‐Creation Process Evaluation

The participatory atmosphere reduced hierarchical barriers, fostering a sense of ownership [69, 70]. Successful co‐creation requires overcoming power inequalities to avoid tokenism [71, 72, 73, 74]. Participant scepticism regarding immediate withdrawal highlights the ‘learned helplessness’ of chronic use, reinforcing that deprescribing is a psychological challenge requiring long‐term empowerment to restore self‐efficacy [75, 76].

## 6 Study Limitations

Beyond anticipated small sample size, an internal participation imbalance was observed during some group dynamics, which required active moderation to ensure all voices were heard. Additionally, time restrictions during the ‘Ideas Laboratory’ prevented every subgroup from evaluating the entire intervention, potentially limiting the overall prioritisation of content.

## 7 Conclusion

The co‐creation process between primary care professionals and clients was essential for designing a realistic, contextualised intervention tailored to the needs of all stakeholders. By aligning with client engagement research principles, this approach fosters more relevant and equitable interventions through active participation.

Co‐creation proved that absolute consensus is not required; instead, it enriches the study framework by negotiating diverse realities through methodological flexibility. This process resulted in a structured eight‐session intervention, complemented by support materials and a nursing training video, providing important considerations for the future implementation and evaluation of the intervention.

## Author Contributions

**Amaia Cuesta‐Zigorraga:** conceptualisation, investigation, methodology, visualisation, data curation, formal analysis, writing – original draft, project administration. **Carlota Las Hayas Rodríguez:** conceptualisation, methodology, validation, supervision, writing – review and editing. **Garazi Aldanondo Aristizabal:** writing – review and editing, investigation, formal analysis. **Clara Sánchez Sánchez:** writing – review and editing, investigation, formal analysis.

## Funding

The authors have nothing to report.

## Ethics Statement

This study was approved by the Research Ethics Committee of the Gipuzkoa Health Area (reference number: CUE‐EMP‐2024‐01). It complies with Law 14/2007 on Biomedical Research, the ethical principles of the Declaration of Helsinki, and all other applicable regulations.

## Conflicts of Interest

The authors declare no conflicts of interest.

## Patient Consent Statement

All participants received written and verbal information about the study and provided informed consent prior to participation.

## Clinical Trial Registration

This study is not a clinical trial and therefore has not been registered in a clinical trials database.

## Permission to Reproduce Material From Other Sources

This manuscript does not contain material reproduced from other sources.

## Data Analysis

The authors affirm that the methods used in the data analyses are suitably applied to their data within their study design and context, and the statistical findings have been implemented and interpreted correctly.

## Supporting information


Supporting File 1

Supporting File 2

Supporting File 3

Supporting File 4

Supporting File 5

## Data Availability

The data that support the findings of this study are available from the corresponding author upon reasonable request. Due to privacy and ethical restrictions, data containing information that could compromise participant confidentiality is not publicly available.
