# Supplementary material for: Development of a Women's Empowerment Nurse‐Led Group Intervention for Benzodiazepine and Z‐Hypnotic Deprescription in Primary Healthcare: Results of a Co‐Creation Process
Source: Health Expect. 2026 Oct 7;29(5):e70895. doi: 10.1111/hex.70895 (PMC13643936; doi:10.1111/hex.70895)
Supplement: Supplementary file 1 [file HEX-29-e70895-s001.docx]

**Additional file 2. Information collection template**

**Activity:**

**Group dynamism**

1. Level of participation

- High
- Medium
- Low

2. Type of participation

- Balanced
- Unequal

3. Quality of interactions

- Collaborative
- Competitive
- Indifferent

4. Flow

- Natural
- Interrupted

5. Level of consensus

- High
- Medium
- Low

**Common answerns:**

**Key interactions:**

**Challenges and obstacles:**

**Annotations:**
