# Supplementary material for: Development of a Women's Empowerment Nurse‐Led Group Intervention for Benzodiazepine and Z‐Hypnotic Deprescription in Primary Healthcare: Results of a Co‐Creation Process
Source: Health Expect. 2026 Oct 7;29(5):e70895. doi: 10.1111/hex.70895 (PMC13643936; doi:10.1111/hex.70895)
Supplement: Supplementary file 2 [file HEX-29-e70895-s004.docx]

**Additional file 1. GRIPP2 short form checklist**

| **Section and topic** | **Item** | **Section in which reported** |
| --- | --- | --- |
| Aim | Report the aim of PPI in the study | 3. Design |
| Methods | Provide a clear description of the methods used for PPI in the study | 3. Design |
| Study results | Outcomes – report the results of PPI in the study, including both positive and negative outcomes | 4. Results |
| Discussions and conclusions | Outcomes: Comment on the extent to which PPI influenced the study overall. Describe positive and negative effects | 6. Conclusions |
| Reflections/critical perspective | Comment critically on the study, reflecting on the things that went well and those that did not, so others can learn from this experience | 5. Discussion |

PPI = Patient and Public Involvement
