# Supplementary material for: Development of a Women's Empowerment Nurse‐Led Group Intervention for Benzodiazepine and Z‐Hypnotic Deprescription in Primary Healthcare: Results of a Co‐Creation Process
Source: Health Expect. 2026 Oct 7;29(5):e70895. doi: 10.1111/hex.70895 (PMC13643936; doi:10.1111/hex.70895)
Supplement: Supplementary file 3 [file HEX-29-e70895-s005.docx]

**Additional file 3. Results of the *Ideas Laboratory* exercise**

| **Session** | **Points** | **Feedback** | **Changes Implemented** |
| --- | --- | --- | --- |
| 1  SITUATING OURSELVES  Part I  Framing the state of self-esteem | 8 | - The exercise is considered a useful approach for participants in the first session. In this regard, it is important to emphasise the need to answer the questions individually. - The questions help you to consider things that you have not questioned until now and invite you to reflect. - It is considered important to give each participant the exercise sheet, which contains the questions, so that they can better follow the dynamics and/or consult them afterwards. | The structure of **Session 1** is maintained. Situating ourselves. |
| 2  SITUATING OURSELVES  Part II  Treatment as a room of one's own | 7 | - They find it very interesting to discover common ground with the other participants, generating feelings such as ‘I am not alone’ or feeling understood. It also creates a sense of group identity. - It is considered important not to force anyone to participate in the exercise, but to let each person ‘experience *it as they wish*’. They offer the possibility of participating by writing on a piece of paper and passing it to the facilitator to read aloud. - They emphasise the value of reflecting on the needs that pharmacological treatment meets, as well as the differences between the issues that are in our hands and those that are beyond our control. - They consider it a useful exercise to raise awareness of pharmacological treatment (‘*you probably haven't thought about it for a long time, without rethinking it*’*).* | The session is eliminated because, in the priority matrix exercise, it has been determined that working directly on pharmacological treatment is not useful. As sessions need to be eliminated, priority has been given to continuing work on other areas that will subsequently influence the medication regimen. However, the need to reflect on the pharmacological regimen and its duration remains. In addition, the value of reflecting on the needs that pharmacological treatment meets is emphasised, as well as distinguishing between issues that are within our control and those that are beyond our control. This issue has been moved to Session 3. My health in my hands. Finally, generating a sense of group cohesion can be achieved through other dynamics. |
| 3  WORKING ON SELF-ESTEEM  Part I  Unravelling the power of what matters to us | 9 | - It helps us reflect on ourselves and, by listening to others, inspires new ideas and personal reflections. - They find the final question difficult (*What are my goals and purposes in participating in this group about my self-esteem?*). They suggest detailing the question or that the moderator provide guidance to encourage reflection and specific answers. - They consider the questions interesting, but some may require more time to answer. They suggest that the facilitator offers examples to facilitate answers to the more complex questions. - They propose changing the order of the questions, starting with what others think of us, as this might be easier than starting with personal reflection. - They suggest adding a quick initial exercise to define what self-esteem means to each participant. | The sessions are merged to create **Session 2**. Self-esteem from a gender perspective, opting for the most effective and highly rated activities: *Discovering the pillars of self-esteem and Reflecting on the social value of masculinity and femininity.* The result is Session 2. Self-esteem from a gender perspective. |
| 4  WORKING ON SELF-ESTEEM  Part II  Gender-based self-esteem | 9 | - They value the gender approach positively, highlighting that there is a lot of talk about self-esteem, but little about gender. - They consider it interesting mainly for older women who have never reflected on these issues, but also for young women, as messages ‘from another era’ continue to be heard today. - They consider it essential to discuss and refine the results of each quality assigned to each gender, as some, although they may seem negative, can have positive aspects (such as sensitivity). |  |
| 5  TOWARDS AN EMANCIPATING HEALTH MODEL  How do I want to take care of my health? | 9 | - They consider it crucial to explain the pharmacological treatment and its effects (as well as the suitability of having it carried out by their referring doctor). Even so, they recognise that there is a lack of knowledge among patients and are unsure whether this is the right place and time to address it. - It is mentioned that withdrawal from the drug causes a lot of fear and that this session could be useful in helping to overcome that fear. - They emphasise the importance of not viewing the inability to withdraw the drug as a failure, suggesting that this aspect should also be addressed. | This session corresponds to **Session 3**. My health in my hands. It focuses on the area of ‘healthcare/self-care’, identified as a priority in the Priority Matrix dynamics. Direct work on pharmacological treatment is avoided, as participants in the co-creation process have assessed it as unhelpful. However, by focusing on an emancipatory model of health, it is hoped that this will have a positive impact on pharmacological guidelines. A key element of the session is reflecting on the importance of not considering the inability to withdraw the drug as a failure. |
| 6  REFLECTING ON GENDER-BASED VIOLENCE  Part I  Making violence visible, its effects and how we respond to it | 10 | - They express doubts about the appropriateness of working on this in a group due to the sensitivity and intimacy of the subject matter (gender violence). - They add that it is also an issue that should be addressed with men. | The sessions are merged to create **Session 4**. Exercises are proposed to reflect on gender-based violence and identify its sociocultural roots, albeit in a more general way, without delving into personal experiences or intimate matters. |
| 7  REFLECTING ON GENDER-BASED VIOLENCE  Part II  Daring to talk about sexual assault, guilt and shame | 10 | - The difficulty of talking about this subject is emphasised, although it is considered entirely relevant in an intervention for female empowerment. |  |
| 8  I AM NOT ANYONE'S BETTER HALF. IN FACT, I THINK I AM A VEGETABLE.  Part I  What myths are part of my story? | 8 | - It is suggested that the reflection on relationships be broadened to include experiences of abuse in other areas, such as children, siblings, or work, which could enrich the debate. - In general, they consider the exercise to be very positive, generating enthusiasm for participation. They highlight that it addresses unusual but important issues. - It is suggested that an overly couple-centric approach be avoided, highlighting the importance of addressing loneliness and how some people fill the void in their lives with children or work, not just romantic relationships. | The sessions are merged to create **Session 5**. The focus on relationships is broadened beyond the couple. The exercises are retained due to their positive evaluation, but the myths that are worked on also cover other areas of relationships (children, work colleagues, friends, etc.). |
| 9  I AM NOT ANYONE'S BETTER HALF. IN FACT, I THINK I AM A VEGETABLE.  Part II  What kind of relationship do I want to build? | 6 | - This dynamic has generated controversy due to its couple-centric approach, although the final task of writing a ‘Farewell letter to my dependent self’ is highly valued. - The question about the desired relationship is questioned, as, for many, it would be more beneficial to leave a relationship. They suggest focusing on ‘relationships in general’ to avoid anxiety and broaden the focus. |  |
| 10  MY BODY IS (NOT) A BATTLEFIELD  How do I relate to my body? | 7 | - The content is considered important, although the proposed dynamic raises doubts. - Although there is a diversity of opinion in the group, it is suggested that some women may feel uncomfortable doing the proposed exercise (lying on the floor and being ‘touched’ by another participant). | **Session 6**. Alternative exercises are proposed to maintain the same objective, without generating conflict (without being invasive or requiring physical contact) and while working individually, which allows each participant to share their experience if they wish, in a more respectful manner. |
| 11  HAVE WE FOUND OURSELVES WITH SEXUALITY? | 4.5 | - The exercise has generated reflection on consent and promiscuity, highlighting that some participants perceive it as more relevant to younger generations, given their limited experience in relationships. - It was noted that, depending on the culture or profile of the woman, addressing issues such as consent and sexuality can be very violent, especially for those who have not prioritised sex in recent years. - It is proposed that the exercise be oriented towards a more observational approach, using videos and debates to encourage reflection, maintaining the poster as a positive element but without exposing or sharing personal experiences. It is suggested that the exercise focuses on listening rather than sharing, focusing on the impact of the relationship with sexuality on the participants’ lives. - One participant said that the questions are monotonous, tedious, and excessively long, perceiving them as a boring task. - It is considered that the issue of violence is not appropriate to address in this setting, either as a group or as professionals. It is suggested that abuse be addressed in other spaces, in a less direct manner, and that the debate be expanded beyond the couple to include abuse at work or by children. | The session will be eliminated due to the low score and negative feedback received. In addition, it has been determined that sexuality is not a priority issue in group intervention, according to the priority matrix. |
| 12  THERE IS MORE THAN ONE MOTHER, and here we are all. Reflecting on the processes of motherhood | 7 | - It is evaluated as a useful and essential session due to the need to highlight motherhood and its effects on emotional well-being. - Using the dynamics and space of the group for ‘exculpation’ is extremely interesting. - As this is a complex and broad topic, it is proposed to work on it in two sessions. It is even suggested to hold one session to delve deeper into motherhood and another session to discuss experiences as a ‘daughter’. - The following topics are highlighted as important: the choice of motherhood, the ideal family, and the concept of the ‘ideal mother’ and the ‘happy family’, seeing the need to demystify the latter. | **Session 7.** The objective of raising awareness of motherhood and promoting the removal of guilt remains, given its relevance. However, following the evaluation carried out during the session, the approach has been rethought to also include reflections from the experience of being a daughter. This expansion seeks to ensure that the session is meaningful for all participants, regardless of whether they are mothers or not. In this new framework, key issues such as the choice of motherhood, the ideal family, and the concepts of the ‘ideal mother’ and the ‘happy family’ are addressed, emphasising the need to demystify these social models. The central objective remains to highlight the influence of the social context in constructing the meaning of motherhood, emphasising how normative ideals affect both their perception and experience. As a new feature, a specific exercise has been incorporated to promote reflection from the perspective of being a daughter, thus broadening the range of experiences addressed in the session. |
| 13  BACK TO THE BEGINNING? Closing processes, achieving goals, taking care of our present, which is our future | x | This session has not been evaluated, as it is a closing session, where the group reflects on the experience and the achievement of the objectives set at the beginning. | **Session 8.** A farewell session is created, where each participant’s self-care goals are evaluated, and exercises are included to close the group. |
